# Co-designing sustainable biochar business models with sub-Saharan African communities for inclusive socio-economic transformation

**DOI:** 10.1038/s41598-024-66120-y

**Published:** 2024-07-09

**Authors:** Ssemwanga Mohammed, Nakiguli Fatumah, Kigozi Abasi, Max Olupot, Morris Egesa, Theresa Rubhara, Aleksandra Augustyniak, Tracey O’Connor, Naoum Tsolakis, James Gaffey, Helena McMahon, Foivos Anastasiadis

**Affiliations:** 1 African Forum for Agricultural Advisory Services (AFAAS), P.O. Box 34624, Ntinda, Kampala, Uganda; 2 Agriculture, Environment and Ecosystems (AGRENES), P.O. Box 5704, Entebbe, Kampala, Uganda; 3 Agriculture, Environment and Livelihoods (AGRILIV), P.O. Box 71257, Makerere, Kampala, Uganda; 4 National Agricultural Research Organisation (NARO), National Livestock Resources Research Institute, P.O. Box 295, Entebbe, Uganda; 5 Circular Bioeconomy Research Group, Shannon ABC, Munster Technological University, Clash Rd., Tralee, Co. Kerry Ireland; 6 Department of Supply Chain Management, School of Economics and Business Administration, International Hellenic University, 57001 Thessaloniki, Greece; 7 Department of Agribusiness and Supply Chain Management, Agricultural University of Athens, 1St Km Old National Road Thiva-Elefsis, 32200 Thiva, Greece

**Keywords:** Sustainability, Environmental impact, Environmental impact

## Abstract

Smallholder farmers in sub-Saharan Africa (SSA) encounter multiple livelihood challenges. Embracing circular bioeconomy principles, particularly considering agricultural and food processing residues, could enable inclusive, locally led, sustainable development pathways within rural communities. Biochar products are one such example of a bio-based material that can be generated using circular principles and deployed for sustainable community development, including among smallholder farmers. This research leverages empirical evidence from four SSA regions to explore the potential of inclusive and sustainable biochar business models, namely: (i) Northern Region, Ghana, (ii) Yamoussoukro, Côte d’Ivoire, (iii) Casamance, Senegal, and (iv) Western Region, Uganda. Co-creation workshops using the Triple-Layered Business Model Canvas framework were carried out in each region with local stakeholders to evaluate the social, ecological, and economic implications of four locally relevant biochar applications: water filtration, biogas purification, soil amendment, and cooking fuel briquettes. Data was analysed at an aggregate level for all regions and applications. The study describes this consolidated biochar business model and examines the implications for SSA communities. The resulting sustainable bio-based business model can guide value chain actors and policymakers in SSA communities towards rural sustainable development with a better understanding of the needs, opportunities, challenges, and impacts of biochar-based value chain development.

## Introduction

Africa’s population has increased by 2.4% per year for the past 30 years and is expected to reach 2.4 billion by 2050^1^. The increase in population poses challenges in terms of demand for resources such as land, water energy, and food. Characterised by much reliance on natural resources, as evidenced by the high volumes of low-value agricultural products dominating the export sector in Africa, the continent is still trailing on many of the United Nations’ Sustainable Development Goal (SDG) targets^2^. In 2022, more than 20% of the continent’s population faced hunger^3^, and one in every three Africans was affected by water scarcity^2^. The shift towards non-renewable energy is relatively slow as most countries still rely on fossil fuel-based energy sources^2^. Another challenge facing Africa will be the impact of climate change. According to the International Rescue Committee^4^, seven of the 10 countries most vulnerable to the effects of the climate crisis are located on the African continent. Droughts caused by changes in the timing and length of rainy seasons are already becoming more frequent. Data from 2010 to 2019 collected from sub-Saharan Africa shows that droughts have almost tripled compared to 1970–1979^5^. Huge areas of the continent are exposed to desertification caused by rising temperatures and changes in rainfall cycles^6^. As 95% of African agriculture is rain-fed^7^, changing climatic conditions threaten the productivity and economic viability of these systems, with negative implications for livelihoods and food security, especially among rural smallholders. African countries and policymakers will have to respond to the need to produce enough food to achieve food self-sufficiency while protecting ecosystems and tackling the effects of changing climate.

A sustainable circular bioeconomy can be a very important tool in the fight against these development challenges, enabling the transformation of bio-based sectors, including the agri-food sector, to support sustainable economic growth and development. Transition to a circular bioeconomy can be achieved through developing circular bio-based supply chains that are internationally coordinated to maximise bio-based resource use, and close and dematerialise resource loops, e.g. through cascading use of biomass and cycling of nutrients to support sustainable biomass production, e.g. in agricultural production systems^8–10^. It can also be a tool to achieve the United Nations’ Sustainable Development Goals (SDGs)^11^. African agri-food systems can contribute to food and nutrition security by alleviating poverty (SDG1) and securing food supplies (SDG2) while supporting inclusive, sustainable rural development (SDG 8). Circular agri-food systems, with an emphasis on efficient resource use and waste reduction, are also an important element for rural African communities to create sustainable livelihoods^12^. In a sustainable community, the available resources are leveraged to meet current demands and improve the quality of life. At the same time, the natural capital, stability, economic prosperity, and social equity of the region are preserved, and sufficient resources are provided for future generations^13,14^.

Another challenge related to the rising population and increasing resource demand, including demands on food production, is the decline in soil quality^15–17^. Biochar has the potential to address this issue^18,19^. This solid by-product of high-temperature heating of biomass in anoxic conditions finds many applications due to its characteristics, stable structure, high porosity and surface area, and the presence of functional groups^20,21^. Biochar can, for example, be utilized as a health-promoting animal feedstock additive^22^, fertility-enhancing soil amendment^23^, solid fuel for energy production^23^, yield-improving biogas additive^24^, or pollutant adsorbent in wastewater treatment^25,26^. Biochar, with its ability to mitigate climate change, contribute to economic development, and enhance livelihoods, has the potential to support the building of sustainable African communities.

Despite the high potential of the bioeconomy in contributing to the SDGs, the transfer of technologies and commercialization of bio-based products such as biochar remains a challenge^27,28^. Though extensive research is available for the uses of biochar, the business models are complex in that biochar is not a uniform product, as its variability is affected by different production conditions such as temperatures, time, pressure, and feedstock type, which in turn affect its use^18,19,22–25,29^. Furthermore, its efficacy is not only determined by the technical properties of the biochar, but also by other conditions of the application in which it is used, for example, the soil properties and environment in which it is applied^28^. Therefore, uncertainty surrounding the biochar properties and its economic viability requires the development of context-specific business models to translate this innovative product into the market. A business model is an abstract concept based on the rationale of the business for creating value, encompassing an organization’s strategies for value proposition, value creation, and value capture^27,30^. To understand the business models and the value created within them, a Business Model Canvas (BMC) can be used as a framework for analysis, to provide a structured aggregate of the business activities, understanding where value is created and which parts may need alterations^31^.

Traditional BMCs are often criticized for their emphasis on profitability and lack of focus on the environmental and social sustainability of their value proposition^32^. The inherent characteristic of circular bio-based business models is that they are formulated to reduce environmental pressure along the product life cycle, increasing efficiency in the consumption of resources and use of renewable raw materials while also incorporating social sustainability^30,33^. When developing the business models, a value chain approach is critical to identify value proposition opportunities and ensure resource efficiency throughout the process from supply of biomass to use of the final product. By considering direct and indirect stakeholders, the social layer of the BMC can be extended. Similarly, environmental aspects are explained using environmental data (e.g., from Life Cycle Assessment) concerning the impact of each value proposition. This extended BMC, known as the Triple Layer Business Model Canvas (TLBMC), is crucial in developing bio-based business models as it provides a more comprehensive lens through which business models can be analysed^33^. Engaging different perspectives of sustainability (i.e. social, environmental and economic), for example by engaging diverse value chain actors and non-value chain stakeholders, can facilitate the development of multi-dimensional business models that take account of local community interests and concerns, and complex system dynamics within and beyond local communities^34^. This study, based on pilot studies in four SSA regions regarding the use of biobased products for smallholder farmers, applies the TLBMC in a co-creation approach with local stakeholders, to develop biobased value chains and unleash the unexploited potential of biochar in agriculture to promote rural development.

## Results

This section presents the sustainable value propositions and triple-layered business models for the biochar production technologies (pyrolysis and hydrothermal carbonization (HTC)) and biochar product lines (soil amendment, solid biofuel, additive for biogas production, and pollution adsorbent for water filtration) that were co-designed with scientists, technologists, and rural stakeholders from the four focus regions: the Northern Region of Ghana, Yamoussoukro region of Côte d’Ivoire, Casamance region of Senegal, and Western Region of Uganda.

A bottom-up approach was employed to develop the Value Proposition Canvas (VPC) and to build the social, economic, and environmental layers of the TLBMC, and provides a better understanding of the key social insights along biochar production and marketing cycles, thereby creating social value and improving TLBMC potential. The co-design process brought producers and customers on a common platform to co-create solutions and generate better ideas with a high degree of originality, end-user value, and improved knowledge of customer or user needs.

### Value proposition canvas for biochar

Table 1 presents the framework of the (VPC). The VPC ensures that biochar products fit market demand and consumer needs by defining customer profiles, including customer jobs-to-be-done, the pains they face when doing their jobs, and the gains accrued after accomplishing their jobs. The VPC also visualizes the most important components of the market value of biochar and how derived product lines relieve pains and create gains for customers (Table 1).

**Table 1:** The co-created Value Proposition Canvas of biochar for biochar products.

Customer jobs	Gain creators	Pain relievers	Gains	Pains
*(i) Soil amendment*				
Sustainable agronomy	Increase soil productivity	Improve soil tilth	High crop yields	Poor soil tilth
Use of organic inputs	Increase crop productivity	Improve soil aeration	Food security	Poor soil aeration
Use of organic fertilizers	Reduced costs of fertilizers	Improve pH and CEC of soil	High incomes	Low pH and CEC of soil
Integrated Soil Water Management	Soil water conservation	Improves water retention	Dry season farming	Poor soil drainage

*(ii) Solid biofuel*				
Training in clean energy	Increase access to bio-briquettes	Clean energy production	Cheap, clean energy	Air pollution from fossil fuels
Provision of inputs for clean energy	Provision of clean energy	Recycling waste into energy	Last-mile access to clean energy	High cost of fossil fuel energy
Switching to bio-briquettes	Reduced costs of energy use	Reduces air pollution	Reliable energy from briquettes	Limited access to electricity

*(iii) Additive for biogas production*				
Training in biogas production	Increase biogas productivity	Clean energy production	Cheap and clean energy	Air pollution from fossil fuels
Provision of inputs for biogas production	Provision of clean energy	Recycling waste into energy	Last-mile access to clean energy	High cost of fossil fuel energy
Switching from firewood and charcoal to biogas	Reduced costs of energy use	Reduces air pollution	Reduces deforestation	Limited access to electricity

*(iv) Medium for water filtration*				
Training in water filtration	Increased access to water	Clean water production	Availability of clean water	Water scarcity
Provision of inputs in water filtration	Provision of clean water	Recycling wastewater	Sustainable water supply	High cost of water
Use of clean water	Reduced costs of water	Reduces water pollution	Access to water for production	Limited access to clean water
Integrated water management	Improved sanitation	Saves time for fetching water	Enhanced Supply of clean water	Time spent fetching water

Table 1 describes the Value Proposition Canvas of biochar for biochar products. This includes the customer jobs associated with each of the four biochar products: soil amendment, solid biofuel, additive for biogas production and medium of water filtration. For each customer job, gain creators, pain relievers, gains, and pains are described.

### Triple layer business model canvas

The TLBMC for the biochar product lines examined has three main layers, namely economic, environmental, and social layers. Therefore, the sustainable TLBMC provides an all-inclusive framework to explore the feasibility and sustainability of economic, environmental, and social impact on commercial biochar production in these four rural SSA regions. The co-designed TLBMC presents a suitable tool to expand the economic-centred approach of traditional business models by developing and integrating environmental and social canvas layers, built from both the lifecycle and stakeholder perspectives, into an extended business model canvas. This TLBMC supports biochar production using a more robust, holistic approach based on sustainability-oriented business model innovation.

#### Economic layer

The economic layer has nine components that describe how the biochar business intends to make money (Table 2). The layer also profiles the customer base, how to deliver value, and the other financing modalities. The “Partners” component profiles the partnerships that biochar businesses could leverage for success during the product life cycle. The “Value Proposition” component represents the value that customers and end-users get from using the biochar product lines. The “Resources” component provides primary inputs and resources for biochar production, processing, and marketing. The “Key Activity” component lists major activities that facilitate biochar production and deliver customer value.

**Table 2:** Economic layer of the Triple-Layered Business Model Canvas for biochar.

Partners	Activities	Value Proposition	Customer Relationship	Customer Segments
Technology (HTC and Kiln) promoters Suppliers of spare parts and equipment Suppliers of inputs (e.g., crop biomass) Research institutions Financial institutions Government agencies District local governments (DLGs) Policymakers Community leaders Off takers/users Academic institutions Farmers and private sector	Technology optimization Collection/Supply of raw materials Eco-production of quality biochar Marketing of biochar products Market to attract and retain clients Research and development Customer relationships Capacity building and Training action	Increased access to eco-products (biochar, biogas, biofertilizers, and water purifiers) Low costs of bio-products Waste recycling into biochar Saves fragile ecosystem Job creation and more incomes Climate action and better carbon sequestration Training of Trainers (ToTs) Clean water and water facilities	Pre-booking system After-sales service Agribusiness training Direct sales Competitive Pricing Supply contracts Product quality traceability	Farmers and farmer institutions Off takers/traders NGOs/CBOs Households Restaurants Schools Universities Hospitals Private sector Retail and wholesale business Government agencies
	**Resources**		**Channels**	
	Physical resources Financial resources Labour force		Customer referrals Mass media Social media	

Costs		Revenues		
Capital investment (e.g., infrastructure) Equipment & technology (HTC & Kiln) Raw materials (e.g., biomass) Labor costs Operational expenses Marketing costs Support facilities (e.g., warehousing)		Direct sale of products (e.g., biochar, biogas, biofertilizers, water purifiers) Equity & seed fund by venture capital/angel investors Marketing services (e.g., warehousing, logistics) Intellectual property rights Consultancy services (e.g., installation & maintenance of equipment) Venture capital and angel investments		

Table 2 describes the economic layer of the Triple-Layer Business Model Canvas for biochar. This describes economic aspects of the business model, including Partners, Activities, Resources, Value Proposition, Customer Relationship, Channels, Customer Segments, Costs, and Revenues.

The “Customer Relationship” component defines the primary relationships between the biochar producers and their off-takers and customers. The “Channels” component is an avenue for reaching out and serving the customers, including the biochar distribution channels and how the value proposition is delivered to the customers. The “Customer Segment” component profiles clientele based on their product preferences, pricing, locations, volume, and quality specifications. The main customer segment in rural Africa is the resource-constrained, subsistence farming communities^35^. The “Costs” component describes cost structures, including primary costs for biochar production and business management. The “Revenues” component describes how biochar businesses generate income by delivering product value propositions to the customer segments.

#### Environmental layer

The environmental layer presents the environmental aspects of the biochar production process and products (Table 3). As such, the layer is informed by the life cycle assessment perspective of the inputs (e.g., crop biomass), biochar production, product use, and waste management, including recycling. The layer explores the environmental benefits deriving from the biochar products, as well as cascading environmental impacts derived from the biochar life cycle. The environmental layer presents the foremost ecological challenges and merits emanating from the production, processing, and use of biochar products and by-products^36,37^.

**Table 3:** Environmental layer of the Triple-Layered Business Model Canvas for biochar.

Supplies and out-sourcing	Production	Functional value	End of life	use phase
Machinery, equipment, and Technology (HTC & Kiln) Production of raw materials (e.g., crop biomass) Supply of utilities (e.g., water & electricity)	Biochar production Provision of support services (e.g., trade, warehousing, ICT) Establish facilities for biochar	Recycling of waste into quality biochar Supply of quality inputs for biogas Regulation of soil pH and tilth Enhanced soil fertility and plant nutrition Enhance soil sink capacity for GHG Water filtration and Supply for use Better public health Better ambient air quality	Recycle crop biomass into biochar Clean energy production Water treatment	Last-mile access to clean energy Low-cost clean energy (biogas) Clean energy to power local businesses Reliable Supply of clean energy Biofertilizers Water filtration and treatment for domestic and industrial use
	**Materials**		**Distribution**	
	Physical materials (e.g., infrastructure) Transport facilities Office/ICT materials		Network of traders Logistics Modes of transport Distances travelled	

Environmental impacts		Environmental benefits		
Climate regulation; Mitigation of greenhouse gas (GHG) fluxes (e.g., CO_2_), Reduced carbon footprint); Improve air quality; Better human health, soil tilth, microbiota, and biochemical profiles; Use of quality water; Clean energy		Improves environmental footprint; Increases soil sink capacity for carbon storage and carbon sequestration; GHG (methane and nitrous) fluxes by providing clean energy; Better soil biology, less soil erosion; Reduced nutrient leaching; Increased Supply of water for production; Enhanced soil fertility and crop yields		

Table 3 describes the environmental layer of the Triple-Layer Business Model Canvas for biochar. This describes environmental aspects of the business model, including Supplies and Out-sourcing, Production, Materials, Functional Value, End of Life, Distribution, Use Phase, Environmental Impacts and Environmental Benefits.

The “Supplies and Outsourcing” component involves key activities in the biochar production process, including processing, value addition, and warehousing. The “Functional Value” describes the output of the biochar production process. The “Materials” component presents key resources in the biochar product life cycle. The “Production” components are key to transforming crop biomass and other inputs into the finished biochar product lines.

The “Distribution” component involves the transportation of finished biochar products. The “Use Phase” encompasses the essential resources that customers deploy to use and maintain the biochar products. The “End-of-Life” component refers to the final phases of the biochar product life cycle and describes how the product end-of-life is managed by the end-users.

The “Environmental Impact” component addresses the environmental costs based on the product life cycle. Besides financial costs, Environmental Impact Assessment (EIA) extends to ecological costs and biophysical indicators^38^. For biochar, the impact indicator matrices are greenhouse gas (GHG) fluxes, biodiversity, and air quality. The "Environmental Benefits” of biochar depend on the design of the production facilities, type of feedstock, pyrolysis temperatures, biochar quality, and output rate^29,39,40^.

#### Social layer

The social layer represents the social pillar of biochar sustainability and relationships between actors at all value chain nodes for the biochar product lines (Table 4). Therefore, the social layer presents the major social impacts emanating from value chain actors' relationships. The “Local Communities” component describes the relationship between the producers, off-takers, suppliers, consumers, and other stakeholders, e.g. policymakers. The “Social Value” defines a corporate responsibility for the producers to focus on how to create benefits for the customers and communities. This component analyses the social value within the producers, even though these organizations may appear to be exclusively profit-driven. The “Employees” are a fundamental component of the social layer, which is concerned with the management of the technical personnel and semi-skilled workforce. “Governance” explains the management structure of biochar producer organizations, including the nature of staff structure and functional specialization, and departmentalization (e.g., marketing, processing, warehousing, ICT, and logistics).

**Table 4:** Social layer of the Triple-Layered Business Model Canvas for biochar.

Local communities	Governance	Social value	Societal culture	End user
Stakeholders (e.g., producers, traders, and suppliers) Biochar business District Local government (DLGs) Farmer institutions Input–output traders and market actors Local agribusinesses (e.g., agri-input dealers, traders, and commercial farmers)	Stakeholder involvement in the product value chain Ownership of business firms Internal organizational structures Shareholding & profit sharing arrangements	Increased incomes Increased energy security Enhance the quality of life through bioeconomy Socio-economic transformation of societies Develop value for farmers and off-takers Restoration of fragile ecosystems Water filtration and treatment Clean water resource Clean ambient air	Sustainable values Societal and cultural spaces (NGOs and CBOs)	End-user segmentation based on socio-economic and demographic profiles
	**Employees**		**Scale of Outreach**	
	Labor force and staff profiles (thus, skills and qualifications) Salient social demographics of staff		Long-term relationships with actors Outreach and impact of actors	

Social impacts		Social benefits		
Social impact metrics such as working air quality, hours, cultural heritage, health and safety, fair competition, community engagement, and respect for intellectual property rights (IPRs)		Social costs, capacity-building training opportunities for the farmers and other end-users, agribusiness opportunities, personal development and community engagement by the staff and other employees, partnership by suppliers, and a resilient bioeconomy		

Table 4 describes the social layer of the Triple-Layer Business Model Canvas for biochar. This describes social aspects of the business model, including Local Communities, Governance, Employees, Social Value, Societal Culture, Scale of Outreach, End User, Social Impacts and Social Benefits.

The “Scale of Outreach” delivers information on the extent and complexity surrounding the relationship between biochar producers and stakeholders, including farmers. This relationship is often based on the medium and long-term social, cultural, and economic interests of biochar businesses. The “End-User” consists of customers or users who produce and consume the value propositions from biochar product lines. Lastly, the “Societal Culture” component embodies possible impacts (either positive or negative) on the biochar production process and firms towards rural African communities.

The “Social Benefits” highlight the capacity of biochar businesses to create social value, such as the creation of job opportunities in the host communities. The “Social Impacts” component describes how biochar businesses impact the community and region within which they procure and process materials and trade their products^32^.

#### Integrating the triple layer business model across the biochar value chain

The co-created biochar products business model has been developed to be circular and responsive to the local social and ecological context. This was achieved through the employment within a co-creation process of the VPC to capture current needs and opportunities that can be addressed by biochar products, and the TLBMC to elaborate the social, ecological and economic aspects of the business models. The VPC provided a valuable initial basis for i) identifying biochar applications of value to the local communities involved and potential market opportunities (Gain Creators, Pain Relievers), and ii) characterising the customer profiles of customers involved in the biochar value chains. These are essential inputs into the economic layer of the TLBMC (Value Proposition, Customer Segments, Revenues). As such, the VPC can be regarded as the cornerstone of the TLBMC. The deployment of the VPC and TLBMC in this research are further described in the “Methodology” section. In this research, the VPC development process also resulted in identification of gains and pain relievers that bring benefits for the broader community, e.g. “reducing deforestation” as a gain associated with improved biogas production with the use of biochar additives, and “reducing air pollution” as a pain reliever associated with solid biofuel use. These address some of the limitations of focusing on “exchange value” between businesses and customers identified by Sparviero^41^, and develop a “social value proposition”, similar to that of Sparviero’s Social Enterprise Business Model^41^. The identification of social and ecological value or “public benefits”, rather than purely commercial value or “private benefits”, is likely a result of the co-creation approach that was undertaken in this research with a diverse stakeholder network of community organizations, public bodies and scientists. A similar relationship between public benefit identification and co-creation with broad stakeholder groups has been observed by Keeys and Huemann^42^. The identification of public benefits that can contribute to sustainable community development in this research underlines the merits of VPC and business model co-creation for identifying value creation opportunities that go beyond financial value creation, and beyond the stakeholders directly engaged in the product value chain. The identification of social value in the VPC, including ecologically-linked social value such as reducing deforestation and air pollution, contributed to the social and environmental layers of the TLBMC (Social Benefits, Social Impacts, Environmental Impacts, Environmental Benefits).

**Figure 1:**
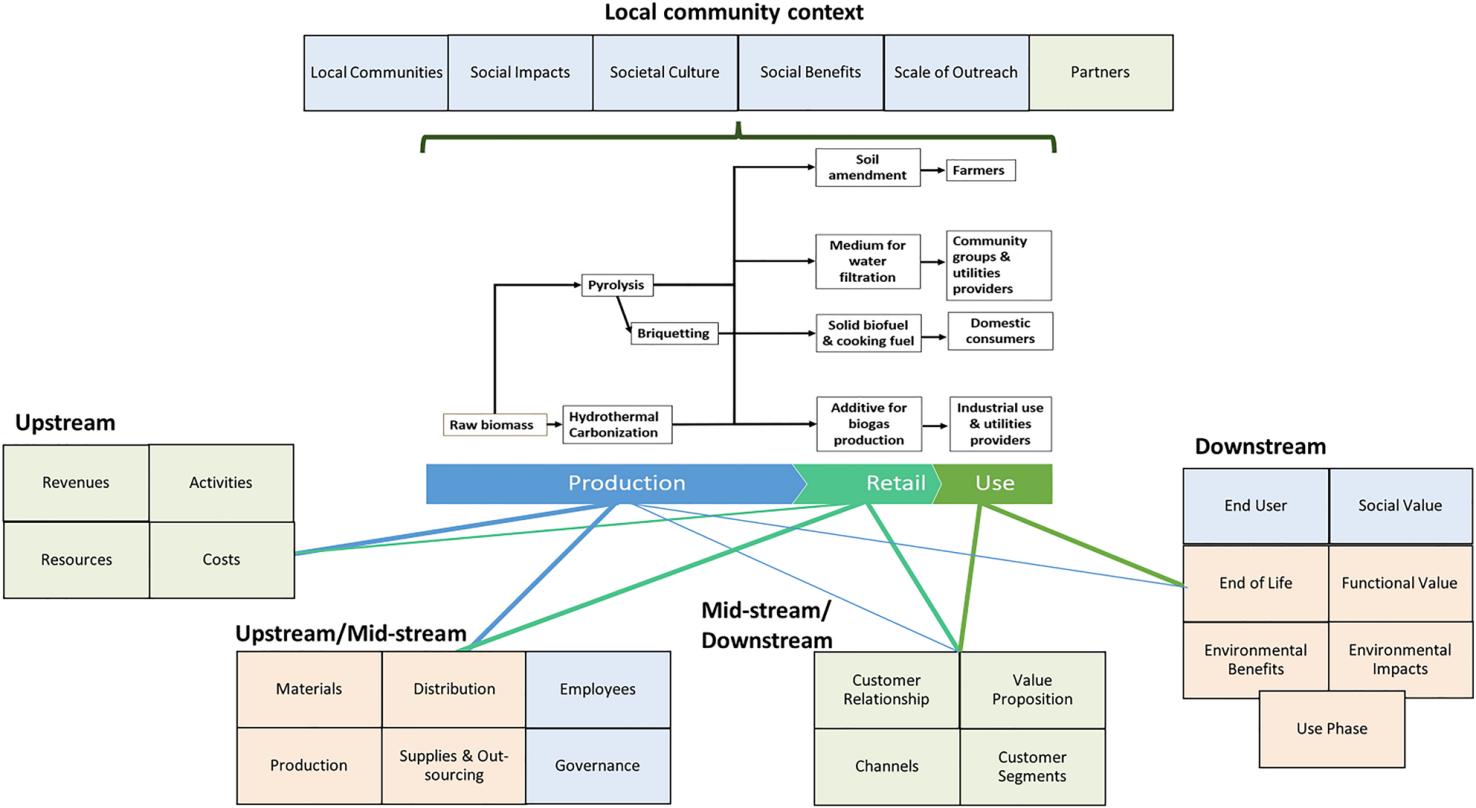
Describes the components of the economic (green), environmental (orange), and social (blue) layers of the Triple-Layered Business Model Canvas, mapped to the value chain stages of the four biochar product lines. This includes Upstream components (Economic layer: Revenues, Resources, Activities and Costs), Upstream/Mid-stream components (Environmental layer: Materials, Production, Distribution, Supplies and Out-sourcing; Social layer: Employees and Governance), Mid-stream/Downstream components (Economic layer: Customer Relationship, Channels, Value Proposition, and Customer Segments), Downstream components (Social layer: End User and Social Value; Environmental layer: End of Life, Functional Value, Use Phase, Environmental Benefits and Environmental Impacts) and the overarching Local Community Context (Social phase: Local Communities, Social Impacts, Social Benefits, Societal Culture, and Scale of Outreach; and Economic layer: Partners).

The deployment of the TLBMC to address social, ecological and economic aspects within and beyond biochar value chains is illustrated by Fig. 1, which maps the components of each layer of the co-created biochar products business model (Tables 2, 3 and 4) to the value chain stages for each of the four product lines. Five of the nine social layer components, and “Partners”, a relationship-focused component of the economic layer, relate to the product value chains but also the local community context beyond the value chain, e.g. governance structures and organisations, Culture, and broader Social Impacts and Social Benefits. Economic aspects related to production enterprise Activities, Resources, Costs, and Revenues are located upstream in the value chain, while economic aspects related to the retailer-customer relationship are located further downstream. Social aspects more concerned with commercial activity, e.g. Employees and enterprise Governance, are also located upstream in the value chain, while End Users and Social Value are located downstream. Environmental aspects are similarly split between upstream/midstream aspects, such as Materials and Distribution, and downstream aspects, such as Use Phase and End-of-Life, reflecting the influence of the life cycle perspective on the environmental layer of the TLBMC. The Environmental Benefits and Environmental Impacts of the four product lines are also most strongly associated with the use stage of the value chain, as the four products generate substantial positive environmental impact and benefits during their use. In the downstream, use-side TLBMC components, there is a circular relationship with production-side activities in the form of return of value to biochar producers (Social Value), and recycling of crop residues and waste into biochar (Functional Value, End of Life).

## Discussion

This research has highlighted the sustainability growth potential, and novel supply chain configuration opportunities, for rural communities in sub-Saharan Africa to further foster equity and welfare, particularly focusing on the case of biochar in regions of Ghana, Côte d’Ivoire, Senegal, and Uganda. The VPC and TLBMC allowed the mapping of the intersection of economic, environmental, and social value emanating from the operationalisation of the four biochar-driven business models investigated.

First, the VPC and TLBMC facilitated the recognition of the operational capabilities of local communities and their capacity to create sustainable impact, in alignment with research efforts on empowerment of local communities for sustainable development^43^. The indicated business potential of biochar and the unique value propositions encapsulated in the commercialisation of the indicated product offerings, promote economic development among rural communities, supporting SDGs 1, 8 and 11, whilst contributing to a range of other SDGs associated with the specific products. The VPC described in Table 1 identifies the contributions of the four biochar products to key resources for sustainable livelihoods, including the availability of, and access to, clean water, clean energy, and food. These essential components of the water-energy-food nexus contribute directly to SDGs 2, 6, and 7^44^. The public health and environmental benefits of the biochar products indicated in the VPC, e.g., reduction in air and water pollution, improved sanitation, and reduction in GHG emissions; also contribute to SDGs 3, 11, 12, and 13^45,46^.

Second, the application of the VPC and TLBMC using a co-creation approach with local and regional stakeholders enabled local communities, researchers, and professionals alike to consider the potential of diverse feedstocks from multiple sustainability perspectives^47^. The empirical evidence gathered through the engagement with community stakeholders from diverse regional settings ensures the validity of the canvas’ content. This novel business model development approach can support biochar commercialisation through engagement of the local community at an early stage, building ownership of the business models and enhancing their viability^48,49^. The holistic sustainability approach of the co-created TLBMC, and resulting insights concerning the business model’s impact on community development, can also inform respective policy-making agendas, at local, regional, and national levels^48^.

Third, this study has demonstrated the social, economic, and environmental implications of biochar exploitation to rural communities in four SSA regions, as perceived by rural community stakeholders. It has also been possible to map the three layers to the stages in the value chain where they have the greatest relevance, which can signpost the most relevant stakeholders to engage for further elaboration of specific TLBMC elements. The understanding of both the community development and business potential of biochar in SSA through the social and economic layers has subsequent implications in terms of equity and fairness in bargaining. The economic layer clearly reveals the income generation opportunities associated with biochar production, processing and retail, and demonstrates the potential for biochar to contribute to SDGs 1, 8, and 9. Utilising crop residues and by-products may be considered a strategic advantage for initiating a range of entrepreneurial initiatives and fostering industrial growth^50^, particularly in developing countries. Leveraging extant agri-based systems provides an opportunity to catalyse the transition towards a sustainable future whilst safeguarding the well-being of rural communities. Due to the circular and participatory nature of the business models developed in the co-creation process, the resulting TLBMC also contributes to addressing SDG 12, as demonstrated by the contribution to both sustainable production and consumption described in Fig. 1. Apart from creating additional income streams for individual farmers, TLBMC can guide the formation of biochar-focused producer organisations, and other enterprises downstream in the value chain, such as biogas and solid fuel providers, that can then help upscale production to develop competitive marketplaces^51^. Specific to the nature of biochar products, and their application for mitigating environmental impacts of human activities, e.g. water pollution, and generating environmental benefits, e.g., soil carbon sequestration, the environmental impacts and benefits are strongly associated with the biochar use-phase. This highlights the specific potential of the biochar products to enable individuals and organisations to address important sustainability challenges, such as those outlined above (SDGs 2, 3, 6, 7, 8, 9, 11, 12, and 13), and create impact within and beyond their local communities.

The co-creation approach in the four regions examined has proved valuable for the development of generalised biochar-based business models for rural SSA communities with strong social and ecological sensitivity, and local validity in the regions where they were developed. The co-created VPC and value chain-mapped TLBMC described in this paper can provide a starting point for other communities in SSA and elsewhere especially those facing water-energy-food nexus challenges, to actively participate in the green transition, including identifying and understanding the opportunities, challenges, and development strategies available to them to deploy and strengthen bio-based value chains in their region, and develop pathways for sustainable community development that are consistent with local needs, resources and socio-ecological context.

### Limitations and recommendations

This study experienced some limitations in its deployment, and from this research specific recommendations for local bioeconomy development research and practice have been developed, namely concerning (i) benchmarking of biochar products against alternatives, (ii) implementation of the TLBMC, and (iii) fostering technological innovation and policy support.

#### Benchmarking biochar products against alternatives

The biochar applications, when benchmarked against alternatives, including bioenergy and other conventional options across different applications, have the following strengths and weaknesses:

i. Soil Amendment: Biochar offers long lasting soil improvement and carbon sequestration but could require a high initial investment, and shows variable effectiveness based on the type and quality of the feedstock. Alternatives like compost and chemical fertilizers provide immediate nutrient release, but chemical fertilizers in particular lack long-term soil health benefits^52^.
ii. Solid biofuel: biochar provides high energy density and carbon–neutral combustion but requires costly, specialized equipment and poses emission concerns. Alternative traditional biofuels, mainly wood and firewood, produce efficient combustion but contribute to the deforestation of woodlots and forest ecosystems^53^.
iii. Additives for biogas production: biochar enhances methane production and digestate quality but also faces compatibility issues and feedstock variability, mainly during the off-season. Alternatives include costly chemical additives and anaerobic co-digestion for biogas enhancement^54^.
iv. Medium for water filtration: high adsorption capacity and sustainability of the biochar make it a cost-effective option for water filtration, but its efficiency and regeneration challenges need optimization. The existing alternatives, activated carbon and synthetic filtration media, have high efficiency but could be cost-prohibitive and have greater associated environmental impacts^55,56^.

In summary, the biochar business models show promise across various applications, particularly where they are more cost-effective or less environmentally damaging than existing alternatives, e.g. chemical fertilizers, traditional biofuels, and chemical biogas additives. However, the product lines require optimization to address weaknesses such as initial investment costs, variability, and regeneration challenges. It was useful to collect data on these strengths and weaknesses in addition to the information generated through VPC and TLBMC co-creation, and benchmark the products against alternatives, in order to contextualize the co-creation outcomes and their implications for sustainable communities.

#### Developing dynamic business models with the triple layer business model canvas

Business model canvases have been criticised for their “static” nature^57^, and therefore failing to account for change or evolution, for example in response to changes in a company’s environment. The TLBMC comprises some components which provide a static snapshot of factors that are subject to fluctuation and can be expected to change, e.g. “Revenues”, “Costs”^32^. The inclusion of the social and environmental layers brings broader perspectives of time and space into the model, compared with traditional business model canvases. This can facilitate the development of models that acknowledge socio-ecological system dynamics and anticipate change. The environmental layer is built on a “life cycle perspective” drawing from life cycle assessment methodologies that take account of the inputs, processes, outputs and environmental impacts occurring at each stage of the life of the product^32,58^. The life cycle perspective brings more diverse temporal, spatial and relational dynamics into the model, compared with traditional business model canvases. The social layer is based on a “stakeholder management approach” to understanding social impact, and is designed to be adapted to specific business contexts, and recognize broader social dynamics beyond the activity of the firm^32^. These layers bring components that cover multiple time horizons, including components that describe factors which are likely to remain consistent over short and potentially medium-term time horizons, e.g. “Materials”, “Governance”, “Functional Value” and “Social Value”, and factors that are likely to remain consistent over longer periods of time, e.g. “Local Communities”, “Societal Culture”. Other components capture short, medium, and long-term aspects within the same component, e.g. “Environmental Benefits”, “Environmental Impacts”, “Social Benefits”, “Social Impacts”. “Scale of Outreach” in particular is designed to characterise business growth trajectories under anticipated scenarios^32^.

In this research, the TLBMC was employed in a co-creation process, and the results from the local co-creation processes were collated to develop generalised business models reflecting salient factors from the different communities involved, described in Tables 1, 2, 3 and 4. The co-creation approach can enhance the relevance of resulting innovations, e.g. business models, to local conditions, including system disruptions and shocks, and therefore improves the likelihood of their adoption, and delivery of positive socio-ecological outcomes^59^. The co-creation process can therefore be a valuable extension of conventional TLBMC application for sustainable business model development and innovation, as demonstrated in this research. As part of the co-creation process, SWOT analysis was included at the value proposition development stage, to encourage collaborators in the co-design process to maintain awareness of external threats and opportunities, i.e. dynamic factors of influence, in addition to the more static internal strengths and weaknesses of the product lines^60^. Cultivating collective awareness of, and developing responses to, potential “opportunity” and “threat” scenarios, or the use of other collaborative scenario planning methods, can support TLBMC application in a way that acknowledges local, regional, and global system dynamics, helping communities to make sense of specific challenges, and develop appropriate strategies^60–62^. To further extend the TLBMC framework to capture dynamics and evolutionary pathways as biochar systems mature, stakeholders should incorporate dynamic components to represent known evolving factors and feedback loops, such as: emerging technologies, market dynamics, regulatory changes, and social and environmental change projections﻿^63^. Integrating phased short or medium-term business model review as an ongoing, iterative scenario planning exercise, e.g. with input from key stakeholders, biochar performance metrics, and updated life cycle inventory and impact information; can also support the viability of business models over time and support communities and businesses to anticipate local, regional and global social, environmental and economic changes, and achieve adaptive organizational learning^61^. The usefulness of the business models under dynamic “real-world” conditions can also be strengthened through ongoing collaborative learning and adaptation within biochar value chains and the local community, e.g. market-based knowledge sharing, pilot and demonstration activities, and experimentation. Incorporating these strategies into the TLMBC roadmap will support stakeholders to navigate complexities, anticipate challenges, and maximize the potential of the biochar value chains for transforming livelihoods and promoting sustainable socio-economic development in rural SSA communities.

#### Moving from co-creation to technological innovation and policy support

The co-creation approach of the TLBMC across biochar value chains involved collaboration between stakeholders to develop business models focused on key product function areas, namely soil amendment, solid biofuel production, biogas enhancement, and water purification. This co-creation approach established a multi-stakeholder platform where stakeholders, including producers, researchers, policymakers, farmers, and off-takers, worked together to tailor novel biochar products and services to their needs. As observed by Keeys and Huemann^42^ a co-creation approach can support eventual benefit realization. However, a benefit capture phase involving key enabling stakeholders, e.g. investors, policymakers, may be needed to achieve realization of sustainable development outcomes^42^. Technological innovation is important in order to optimize the applications, while policy support is required to incentivize adoption and scale out innovations^64^. The next phase of this research will focus on technological improvements, including improving biochar formulations and applications to optimize the identified product lines and deliver on their potential to enhance soil fertility, energy production, anaerobic digestion, and water quality. The process of bringing these innovations to the attention of local, regional and national policymakers and advocacy organizations has already begun, with some of these actors playing a role in the co-creation workshops, which can help shape a joint agenda and build policy influence^65^. The process of building policy support will continue during the next stages of this research, e.g. communication of policy briefs describing the research findings, and beyond the lifetime of the project through the multi-stakeholder platform that has been developed during this research.

Sustainable communities make effective use of natural resources, enhance the environment and strengthen economic resilience, using socially inclusive approaches and accounting for the needs of present and future residents and other resource users^66^. Lennon and Dunphy^67^ highlight the centrality of social sustainability for achieving sustainable communities, while social inclusion is recognized as a core dimension of social sustainability^68^. The integrated strategy described in this research, engaging local communities and natural resource users in an inclusive, co-creation approach, and supporting these communities to achieve greater sustainability through technological innovation and policy advocacy, can result in sustainable, realistic, and effective solutions for managing challenges experienced by rural SSA communities. The co-creation and innovation approach in particular engages the local community in the development of novel, circular, bio-based business models, and encourages policy action and technological innovation, with potential knock-on effects for fostering community sustainability^66–69^.

## Methodology

### The biochar products

Agricultural biomass and residues such as peanut shells, cashew shells, millet, corn stalks, and rice husks were the raw materials investigated for producing the four biochar products, namely biochar as a product for: (i) soil amendment, (ii) solid biofuel, (iii) additives for enhancing biogas production, and (iv) as a medium for water filtration. Traditional and contemporary kilns and hydrothermal carbonization (HTC) were proposed to produce biochar as a soil amendment product, as a medium for water filtration, and as additives for enhancing biogas production. These technologies in combination with a briquetting line were proposed to produce briquettes for use as biofuel. To achieve product-market fit, biochar value propositions were adjusted based on market intelligence data and insights from customers.

### Data collection

Primary and secondary data were collected using both qualitative and quantitative methods. Primary data was obtained directly from the field through observation, documentation, and in-person interviews with participants during surveys, stakeholder meetings, and workshops. Secondary data in the form of literature and product statistics were collected from desk reviews of published materials. The data collection techniques used were observation techniques, in-depth interview techniques, and documentation of the product lines. The in-depth interviews and focus group discussions were used to provide data on biochar applications and input into the commodity VPC and TLBMC.

Frameworks and tools to evaluate the feasibility of novel business models following the circularity principles have been developed, with SWOT analysis (strengths, weaknesses, opportunities, and threats) being recognized as helpful in informing pertinent research across diverse fields, and identifying prospective future scenarios^60^. Indicatively, SWOT analysis has been used to systematically guide the application of novel technologies, such as Artificial Intelligence, for enabling circularity in the building construction industry^70^. In addition, a SWOT analysis has been carried out to establish a consensus on the concept of sustainable organic solid waste management via the application of the Circular Economy^71^. The SWOT analysis techniques were used in this research to profile key actors and value chain nodes of the biochar product lines, including the strengths for future business development, weaknesses to be averted, and opportunities and threats associated with business development. The data were used as an input in co-designing value propositions and business models for the biochar product lines.

### Co-designing the value propositions

**Figure 2:**
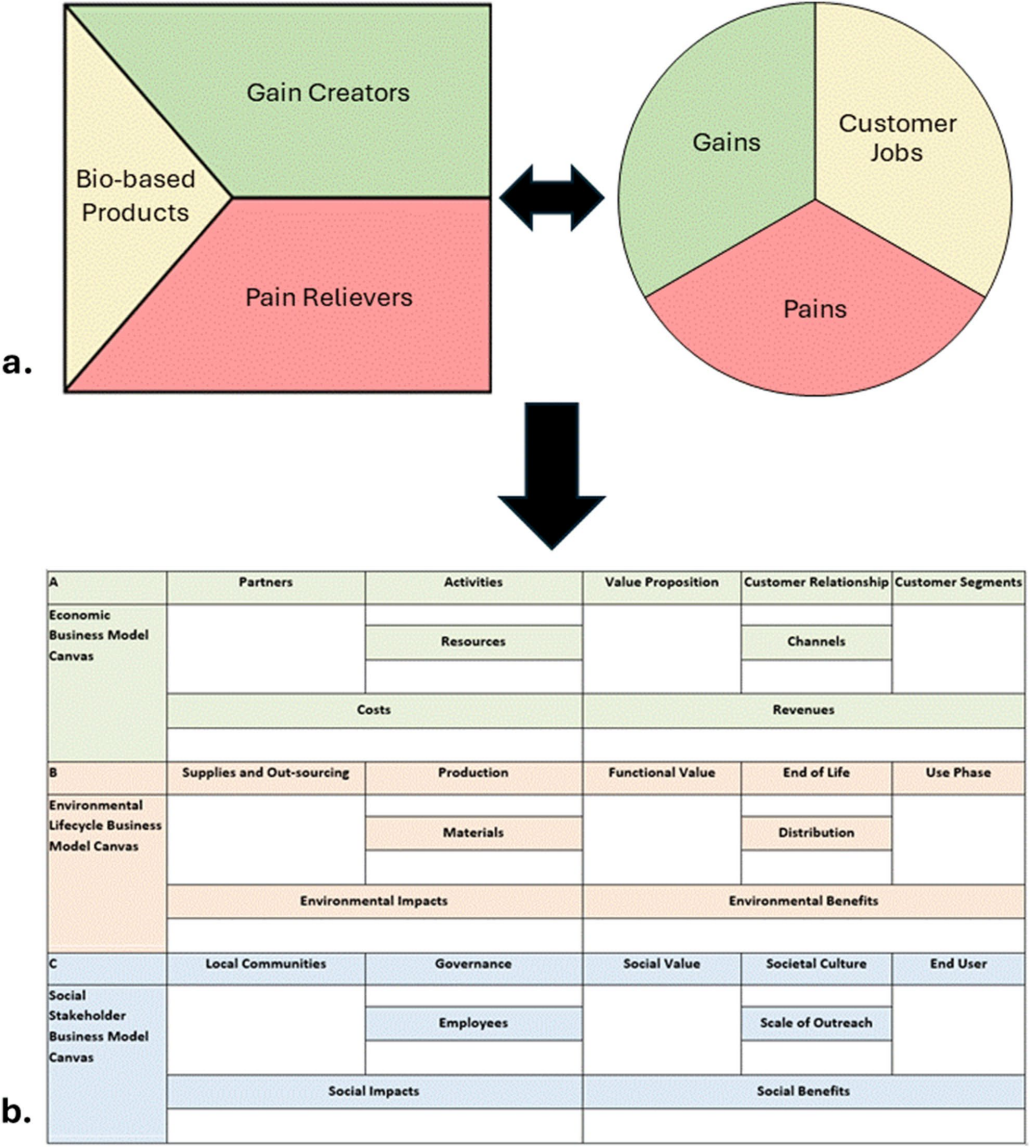
Schematic depiction of (**a**) the Value Proposition Canvas tool and (**b**) the Triple Layer Business Model Canvas. (**a**) describes the schematic depiction of the Value Proposition Canvas tool used, including product line characteristics, gain creators, and pain relievers, and customer jobs, gains and pains. (**b**) describes the schematic depiction of the Triple Layered Business Model Canvas, including the economic layer with the Partners, Activities, Resources, Value Proposition, Customer Relationship, Channels, Customer Segments, Costs, and Revenues components; environmental layer with the Supplies and Out-sourcing, Production, Materials, Functional Value, End of Life, Distribution, Use Phase, Environmental Impacts and Environmental Benefits components; and the social layer with the Local Communities, Governance, Employees, Social Value, Societal Culture, Scale of Outreach, End User, Social Impacts and Social Benefits components.

Multi-stakeholder meetings and workshops were conducted in Yamoussoukro (Côte d’Ivoire), Northern Region (Ghana), Casamance (Senegal), and Western Region (Uganda) to engage stakeholders, mainly farmers, policymakers, scientists, community leaders, and other commodity value chain actors. The actors provided data and were engaged in the co-designing of value propositions based on the VPC template (Fig. 2a).

During the co-design process, the participants developed value proposition profiles for bio-based products, specifically biochar product lines, including their purpose, features and product description, gain creators (e.g. features that create more money, time or provide ease of access), and pain relievers (e.g. features that create solutions, address problems or raise thresholds). Alongside the value proposition profiles, they developed customer profiles describing the customer's jobs (i.e. steps the customer has to take to learn and use the products), gains (e.g. desires, benefits, time-savers, money-savers), and pains (e.g. problems, inconvenience, annoyances) (Fig. 2a). This contributed directly to the development of the Triple Layer Business Model Canvas (Fig. 2b) for the co-design of sustainable business models, further described in section “Co-designing sustainable business models”.

### Co-designing sustainable business models

Commodity value chain actors were engaged in a workshop to co-design sustainable business models for the four biochar product lines that value proposition profiles had been developed for, in multi-stakeholder meetings and workshops, as described in the unidirectional relationship between Fig. 2a and b. The business models acknowledged social and environmental factors and supported sustainable development objectives. They were co-designed using the TLBMC (Fig. 2b), a business model canvas variant that is a modern tool for analysing the economic, environmental, and social aspects of the bio-based products based on customer and stakeholder perspectives^32^. The TLBMC has three layers, namely the economic, environmental, and social layers (Fig. 2b). The economic analysis layer of the TLBMC focused on the analysis of nine major components, namely the customer segments, customer relationships, channels to the customers and end-users, revenue streams, key activities, resources, partners, cost structure, and value proposition. By utilizing the TLBMC template, the initial value propositions were elaborated into more nuanced business models that accounted for economic and social impacts, and data were generated for clientele, markets, and revenue streams, as well as the environmental and social benefits of the biochar product lines.

### Ethical approval

Data was collected through in-person interviews with participants. No experimental procedures were used in this research. All data collection methods were carried out in accordance with relevant guidelines and regulations, including those defined as part of the Ethics Appraisal Procedure of the Horizon 2020 Framework Programme for Research and Innovation, through which this research was funded (GA Number 101000762). The data collection protocols were developed by the African Forum for Agricultural Advisory Services (AFAAS), in line with the relevant guidelines and regulations, and approved by the Uganda National Council of Science and Technology (UNCST). Informed consent was obtained from all participants in the co-creation workshops.

## Data Availability

The datasets generated during and/or analysed during the current study are available from corresponding author Semwanga Mohammed on reasonable request.
