# Author Correction: Co-designing sustainable biochar business models with sub-Saharan African communities for inclusive socio-economic transformation

**DOI:** 10.1038/s41598-024-70051-z

**Published:** 2024-08-21

**Authors:** Ssemwanga Mohammed, Nakiguli Fatumah, Kigozi Abasi, Max Olupot, Morris Egesa, Theresa Rubhara, Aleksandra Augustyniak, Tracey O’Connor, Naoum Tsolakis, James Gaffey, Helena McMahon, Foivos Anastasiadis

**Affiliations:** 1 African Forum for Agricultural Advisory Services (AFAAS), P.O. Box 34624, Ntinda, Kampala, Uganda; 2 Agriculture, Environment and Ecosystems (AGRENES), P.O. Box 5704, Entebbe, Kampala, Uganda; 3 Agriculture, Environment and Livelihoods (AGRILIV), P.O. Box 71257, Makerere, Kampala, Uganda; 4 National Agricultural Research Organisation (NARO), National Livestock Resources Research Institute, P.O. Box 295, Entebbe, Uganda; 5 Circular Bioeconomy Research Group, Shannon ABC, Munster Technological University, Clash Rd., Tralee, Co. Kerry Ireland; 6 Department of Supply Chain Management, School of Economics and Business Administration, International Hellenic University, 57001 Thessaloniki, Greece; 7 Department of Agribusiness and Supply Chain Management, Agricultural University of Athens, 1St Km Old National Road Thiva-Elefsis, 32200 Thiva, Greece

Correction to: *Scientific Reports* 10.1038/s41598-024-66120-y, published online 09 July 2024

The original version of this Article contained an error in Figure 1, where it was erroneously indicated that biochar from hydrothermal carbonization was a constituent material in biochar-based briquettes that were examined in the course of this research.

The original Figure 1 and accompanying legend appear below.

**Figure 1:**
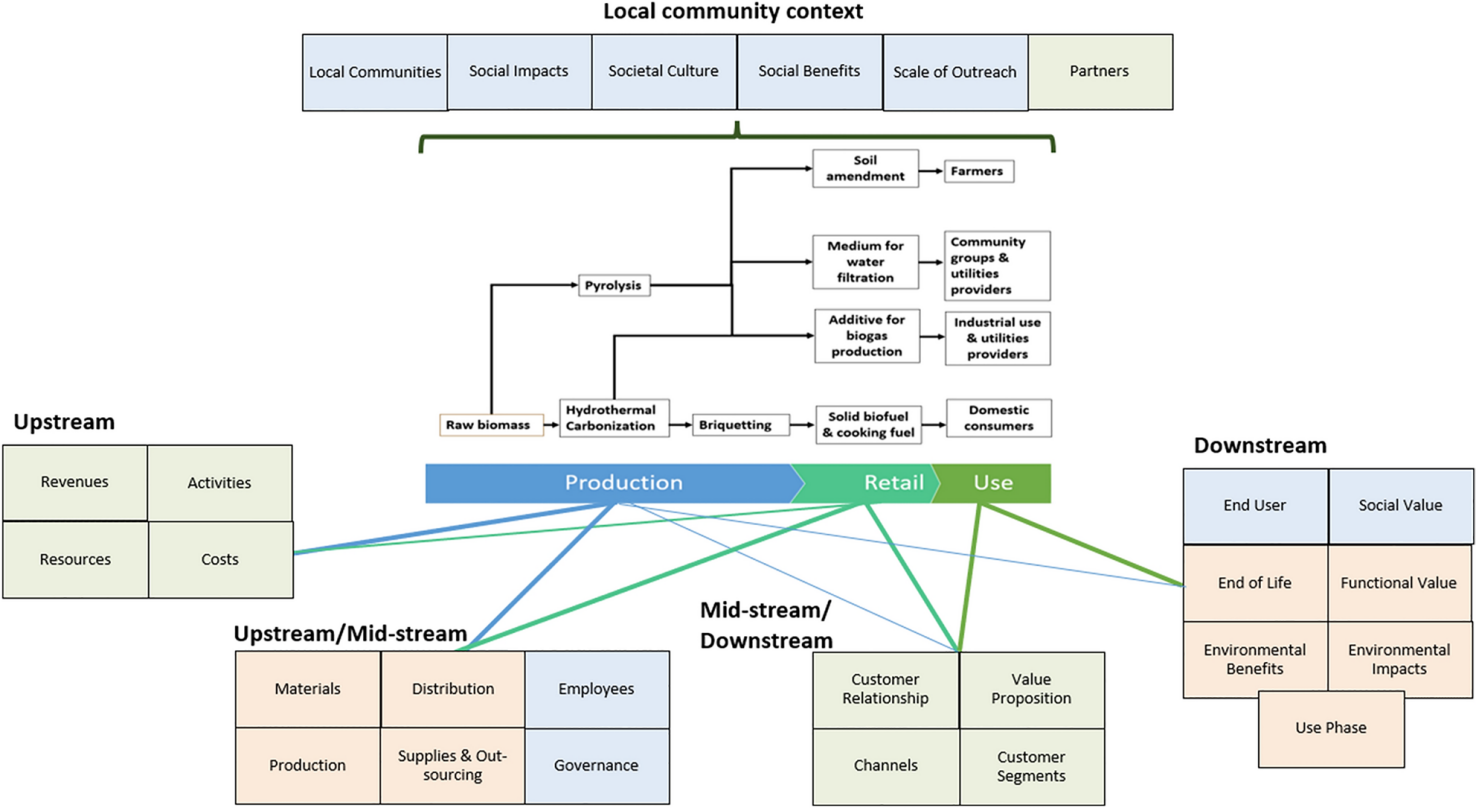
Describes the components of the economic (green), environmental (orange), and social (blue) layers of the Triple-Layered Business Model Canvas, mapped to the value chain stages of the four biochar product lines. This includes Upstream components (Economic layer: Revenues, Resources, Activities and Costs), Upstream/Mid-stream components (Environmental layer: Materials, Production, Distribution, Supplies and Out-sourcing; Social layer: Employees and Governance), Mid-stream/Downstream components (Economic layer: Customer Relationship, Channels, Value Proposition, and Customer Segments), Downstream components (Social layer: End User and Social Value; Environmental layer: End of Life, Functional Value, Use Phase, Environmental Benefits and Environmental Impacts) and the overarching Local Community Context (Social phase: Local Communities, Social Impacts, Social Benefits, Societal Culture, and Scale of Outreach; and Economic layer: Partners).

The original Article has been corrected.

